# Ensuring equity with pre-clinical planning for chlamydia vaccines

**DOI:** 10.1038/s41541-023-00726-7

**Published:** 2023-09-06

**Authors:** Amanda L. Collar, Tegan N. Clarke, Andzoa N. Jamus, Kathryn M. Frietze

**Affiliations:** Department of Molecular Genetics and Microbiology, School of Medicine, University of New Mexico, Albuquerque, NM USA

**Keywords:** Medical research, Vaccines

## Abstract

*Chlamydia trachomatis* (Ct) remains the most common bacterial sexually transmitted pathogen worldwide, causing significant morbidity particularly among women, including pelvic inflammatory disease, ectopic pregnancy, and infertility. Several vaccines are advancing through pre-clinical and clinical development, and it is likely that one or more vaccines will progress into human efficacy trials soon. In this Perspective, we present a case for considering the challenges of Ct vaccine development through a lens of equity and justice. These challenges include the need to protect against multiple serovars, in both females and males, at multiple anatomic sites, and in resource poor areas of the world. We propose that early consideration of vaccine implementation by conducting community-engaged research will ensure that a scientifically sound chlamydia vaccine promotes equity, justice, and shared-gendered responsibility for STI prevention.

## Introduction

*Chlamydia trachomatis* (Ct) is the causative pathogen of the most common bacterial sexually transmitted infection worldwide. Repeated or prolonged Ct infection can cause serious medical sequelae among women, causing pelvic inflammatory disease, ectopic pregnancy, and infertility due to the inflammation and damage to the fallopian tubes caused by ascending bacterial infection^1–3^. High rates of asymptomatic infections coupled with shortcomings in current screening protocols further increase the risk of prolonged, undetected infection. Further, infection with Ct can put women at risk of acquisition of additional STIs and worsening outcomes, such as progression of cervical malignancy in women co-infected with HPV and Ct^4–8^. Additionally, national mass screening programs and consequent treatment of identified cases has had little impact on incidence of Ct. Because of the significant morbidity, associated medical costs, and the ineffectiveness of national mass screening programs, both the World Health Organization and the National Institute of Allergy and Infectious Disease have highlighted the need for research that could result in an efficacious Ct vaccine^9,10^. Indeed, numerous laboratories have undertaken research to engineer a prophylactic vaccine to prevent Ct infection and the associated morbidity^11,12^.

The difficulty of developing a Ct vaccine is highlighted by the decades-long efforts that have been undertaken to reach this goal. However, as we have recently observed with the challenges of the COVID-19 vaccine efforts, it isn’t enough to only develop a highly efficacious vaccine—considerations of delivery to vulnerable populations, cost, and patient acceptability are as important for the success of a vaccine. In this Perspective, we discuss lessons learned from historical vaccine development and opportunities for a patient-centered approach toward Ct vaccine design that may better promote acceptability and, ultimately, equity and justice. By adding an equity lens to the Ct vaccinology research currently being undertaken at the pre-clinical phase, we can work toward ensuring the success of a future Ct vaccine. In the next sections, we discuss potential pitfalls that are likely to impact Ct vaccine implementation and success including: multiple Ct urogenital serovars, gender-based vaccination, consideration of multiple infection sites, and delivery to resource poor areas. We conclude by calling for community-engaged research to be conducted to guide patient-centered vaccine design.

## Protection against multiple *Chlamydia* serovars will promote vaccine equity

At least 11 distinct urogenital Ct serovars exist, which are defined by differences in the amino acid sequences of the major outer membrane protein variable domains I-IV^13,14^. Clinical treatment of urogenital Ct does not differ based on infecting serovar and, thus, serotyping is rarely undertaken in the clinical setting. Ct serovars D, E, and F are the most prevalent circulating serovars, which account for 60–70% of urogenital infections^15–20^. Additionally, there are 3 lymphogranuoma venereum (LGV) serovars of Ct that primarily infect men who have sex with men (MSM) but can also infect women. Infection with the LGV serovars can be particularly severe, (include symptoms). Therefore, although an ideal Ct vaccine would provide protection against all urogenital and LGV serovars, many pre-clinical Ct vaccines focus on protecting against these more prevalent urogenital serovars^21–23^.

Studies suggest that Ct serovars may have differential pathogenicity and rates of transmission. Indeed, a longitudinal, nested case-control study aimed to identify serovar-specific risk for cervical squamous cell carcinoma (SCC) in women with human papillomavirus found that women with Ct serovar G infection were 6.6 times more likely to develop SCC^24^. They also found associations with serovars I (Odds Ratio, 3.8) and D (Odds Ratio, 2.7)^24^. Additionally, serovars F and G are more commonly associated with lower abdominal pain in women than other serovars^25^. Another study confirmed this finding, demonstrating that serovar F was associated with symptomatic, severe endometrial disease and PID^26^. Alternatively, Dean, et al. found that infections with serovar E were associated with mild infections or asymptomatic disease^26^. If a vaccine were to only protect against the most prevalent Ct serovars, the field may be missing an opportunity to provide protection against more severe disease. Indeed, protection against additional urogenital serovars may reduce morbidity among women, since serovars other than D-F may be more commonly associated with SCC, PID, and severe disease^24–26^. Likewise, inclusion of LGV serovars in a vaccine could expand protection to MSM.

There have also been epidemiological studies undertaken that describe differences in urogenital serovars detected among people of different self-reported races. For example, among patients presenting at an STI clinic in Birmingham, Alabama, there was an association between serovar Ia infection and self-reported Black race^27^. If Ct serovars truly are differentially distributed among populations, then advancing a Ct vaccine that protects against all Ct serovars becomes a matter of social justice.

Focusing vaccine efforts on only a few pathogenic strains that circulate in majority groups can have major consequences for the usefulness in marginalized groups. We can learn from the development and implementation of the pneumococcal vaccine (PCV-7). PCV-7 was designed to provide protect against serotypes 4, 6B, 9 V, 14, 18 C, 19 F, and 23 F, which results in a coverage of 90% of the pneumococcal serotypes circulating within the United States and Canada^28^. However, this coverage is dramatically reduced in places like Africa (67%), Latin America (63%), India (53%), and Asia (43%)—areas where pneumococcal infection is more devastating due to limited access to medical care^28–33^. Likewise, Gardasil 9 aims to protect against 7 high risk HPV types 16, 18, 31, 33, 45, 52, and 58 that are known to be associated with cervical cancer. However, among Native American/Indigenous women, HPV 16 was not the most prevalent high risk HPV isolate, as compared to that reported globally; instead HPV 51 was the most prevalent, which is not targeted by Gardasil 9^34–36^. We can learn from these historical lessons by advancing Ct vaccines that provide cross-protection for all urogenital serovars. Utilizing vaccine platforms that are amendable to mixed formulations, targeting epitopes with shared homology and function between serovars, and engineering vaccines that target multiple antigens and mediate cross-serovar protection are reasonable means to providing more extensive protection. This has been observed with the HPV vaccine, which may provide cross-protection to related HPV strains not included in the formulation, reducing the prevalence of non-vaccine targeted genotypes^37,38^.

## Gender inclusive vaccination will lead to shared responsibility for prevention of chlamydia

A study of newsprint articles within the UK found that males are often absent from articles reporting on teenage pregnancy, abortion, or contraception, demonstrating a lack of shared gendered responsibility for such issues^39^. Indeed, studies show that women are perceived to be more sexually responsible; women may be expected to stay abstinent, promote the use of condoms, utilize hormonal contraception, undergo regular STI testing, and be more knowledgeable about STIs than their male counterparts^39–45^. Additionally, the responsibility of preventing STIs and pregnancy largely falls on women. The Centers for Disease Control and Prevention recommend that sexually active women under the age of 25 or those over 25 at increased risk be tested for Ct infection annually^46^. Despite equally high transmission of Ct from male-to-female and female-to-male^47^, men who have sex with women (MSW) are not recommended to undergo the same rigorous screening^46^. However, annually screening heterosexual men for STIs would likely lead to a decline in Ct infection among their female partners, as heterosexual women are infected by their male partners.

The burden of vaccination against HPV, which was initially only approved for administration in females aged 9–26, has fallen on women^48^. On September 9, 2009 the Food and Drug Administration advisory panel recommended approval of Gardasil for males 9–26, with routine recommendation for males 13–21 occurring in 2011^48^. This did not go without criticism though, with some citing that improved coverage among girls would alleviate the need to vaccinate boys and others demonstrating that only under the most favorable assumptions would adding males be cost effective^49,50^. According to the National Health Interview Survey, women were more likely than men to have received one or more doses of the HPV vaccine across all survey years (2013–2018)^51^. In 2018, only 27% of males had received at least one dose of the HPV vaccine compared to the nearly doubled amount of females at 53.6%^51^. This is a burden placed unequally on girls that is not without risk or consequence; 2–3 vaccinations over 2–3 doctor’s appointments. They may experience associated pain at the injection site, fever, dizziness, nausea, and fatigue. They may miss school days and important lessons during these appointments or because of the vaccine side effect profile.

Current vaccine efforts for Ct focus almost exclusively on preventing infection of the female reproductive tract, and all indications suggest that human clinical trials of Ct vaccines will be conducted in women, with men possibly included after initial approval, like the HPV vaccines. Such a progression for Ct vaccines will continue the status quo of placing the burden of STI prevention on females. To ensure equity and an ethical shared responsibility for STI prevention, steps can be taken now to develop and test vaccines for males in the pre-clinical stage of Ct vaccine development.

## Protection at multiple infection sites will make vaccination inclusive of all people

Infection, pathology, and immunity of sexually transmitted Ct have almost exclusively been studied in the female reproductive tract. However, sexually transmitted Ct infections are not limited to this anatomical site. Individuals engaging in vaginal, anal, or oral sex with an infected individual can become infected in the vagina, anorectum, or oropharynx, respectively, regardless of their or their partner’s biological sex. Additionally, few animal models have been validated to study sites of Ct infection other than the female urogenital tract. This, along with a lack of detailed understanding of the natural history of Ct infection in men and women who engage in various sexual behaviors, means that focusing vaccination strategies on preventing female reproductive tract infection alone will exclude many people from the potential benefits of Ct vaccination. An ideal Ct vaccine would prevent infection at all anatomic sites that are susceptible, to prevent disease as well as transmission. Below we discuss Ct infection at these additional sites.

### Male urogenital tract Ct infection

Studies in Ct infection and immunity in males have not been as robust nor as common as in females, as severity and morbidity of infection are minor in comparison. Further, roughly half of male infection cases are asymptomatic, limiting detection of infection, particularly in light of CDC recommendations against routine screening of heterosexual men^52^. Urogenital infection in males can lead to urethritis, epididymitis, epididymo-orchitis, and prostatitis^53–57^. All these conditions can lead to tissue damage and scarring during chronic infection. Prostatitis, in particular, can occur in the absence of a urethral infection^56,57^. It has been hypothesized that Ct prostate infections may cause inflammation and impair normal functionality of the gland and, thereby, impact male fertility^58^. Yet, the correlation between male Ct infection and infertility is not widely accepted, with contradictory research findings^59–63^.

It is not well understood why males have lower rates of morbidity in urogenital Ct infections. The structure of the male reproductive tract is thought to play a role, as the male has a longer urethra, which lends to bacteria needing to travel much farther to infect additional tissues. Even if severity of infection and resulting morbidity is lessened in males, there are still unpleasant medical consequences to infection, including penile discharge, dysuria, and pain and edema to testicles. Additionally, males are also able to transmit infection to their sexual partners. Therefore, developing a Ct vaccine that protects against infection in both the male and female reproductive tracts would be ideal, as this would reduce morbidity among males and females, and reduce transmission to others.

Indeed, as argued by Hull et al. men play a critical role in transmission of STIs and vaccination of men against HPV (or perhaps other STIs like Ct) is vital in promoting women’s health and achieving herd immunity^64^. When vaccination also poses minimal risk to males and may provide direct benefit through prevention of morbidity, it becomes a strong case to vaccinate all patients and pursue gender-inclusive vaccine policies^64^.

### Anorectal Ct infections

Anorectal Ct infections can arise in individuals regardless of biological sex even if they do not engage in receptive anal intercourse. Anorectal screening is typically recommended only for men who have sex with men (MSM), even though women may also be at risk^65^. Indeed, up to 30% of women in the U.S. have engaged in anal intercourse. Additionally, repeat infections at this site are common and are thought to be multifactorial. They may be due to resumption of sex after treatment or autoinoculation from the rectum back to the vagina (or vice versa) in women due to the close proximity of these sites^66^. This may mean that protection at the anorectum is important for females, even those who do not engage in anal receptive intercourse.

Complications from anorectal infections can be categorized into two types: L- and non-L serovars. Non-L serovars (D-K) typically elicit anorectal infections that are asymptomatic, but fever, proctitis, colitis, and nonspecific inflammation can arise. L serovars (L1-3), though not the focus of this manuscript, typically elicit more severe complications than serovars D-K, including severe inflammation, anal ulcers, fistulas, rectal abscesses, structuring, granulomas, lymphadenopathy, elephantiasis, hematochezia, and friable mucosa^67,68^.

These anorectal infections can also localize only to the anorectal cavity, sparing the urogenital tract completely. Indeed, in one study of MSM, 53.5% of chlamydial infections were within in the rectum alone^69^. Thus, an individual may screen negative for chlamydia with a urine sample, but may have active anorectal infection, necessitating a high index of suspicion to test via anal swab. Eliciting protection at this site is important, as it would prevent transmission to others, and would prevent infection progression to more severe morbidities. In various preclinical models, it has been found that gastrointestinal (GI) immunity alone is not capable of clearing anorectal Ct infection, which would pose a challenge for vaccines^70^. This may call for investigating alternative routes of administration, such as an intranasal spray or oral administration, to increase the immune response in the GI tract beyond what is elicited by infection. Developing a vaccine that is effective at this site would be beneficial to all sexually active individuals. And, since LGV serovar infection is common among MSM, inclusion of LGV serovars in Ct vaccine development becomes an issue of inclusion and equity.

### Oropharyngeal Ct infections

Oropharyngeal Ct infections arise in those who engage in oral intercourse. There was no difference in the prevalence of oropharyngeal infections between MSM (median 1.7%), MSW (median 1.6%), and women (median 1.7%)^71^, demonstrating sexually active persons are at risk regardless of their sexual orientation. Infections at this site are typically asymptomatic, but are capable of infecting urogenital and anorectal tissues when oral sex is performed^72,73^. Yet, there is no evidence that Ct is spread through mouth-to-mouth contact. Developing a Ct vaccine that protects against oropharyngeal infection would further limit a possible source for infection to more vulnerable tissues, where more severe morbidities can arise.

## Advancing vaccines that can be distributed to resource poor areas will ensure vaccine justice

Between 75 and 85% of the estimated 376 million new cases of the four curable STIs (gonorrhea, chlamydia, syphilis, and trichomoniasis) occur annually in low-income and middle-income countries/developing countries^74^. For example, Sub-Saharan Africa, along with other developing areas of the world, still undertake a syndromic approach to Ct infection, rather than implementing screening and control programs^75–79^. This results in Ct infections and the associated morbidity placing a substantial burden on these countries^78^. The prevalence of Ct infection among women of reproductive age in Sub-Saharan Africa is estimated to be 7.8%, which is higher than the global estimated prevalence^78^. This is even higher among HIV positive women^80^. Further, this could be a vast underestimate, as many women experiencing asymptomatic infection may not be diagnosed via the syndromic approach^78^. These unidentified infections substantially increase the risk for morbidity in these women and their children in the case of antepartum infection.

Preferred vaccine characteristics for any Ct vaccine advanced to development must include feasibility of use in low-resource settings where Ct prevalence is the highest. Utilizing vaccine platforms that eliminate the necessity for cold-chain or other special handling during vaccine transport and storage could drastically improve vaccine equity and ensure delivery to resource poor areas of the world. As recently demonstrated by inequities in COVID-19 vaccine distribution, underlying structural obstacles to equitable vaccine distribution, including the disproportionate number of vaccine developers and manufacturers located in high-income countries, exacerbate difficulties posed by availability and storage and distributive capacity in low-income countries^81,82^.

Access to vaccine development and production (sometimes termed “vaccine sovereignty”) is highly limited by global socioeconomic barriers. Few financial incentives exist in the free market for pharmaceutical and biotechnology companies to invest in vaccine research and development^83^. Despite their cost efficiency and enormous public health benefit, the revenue potential for vaccines for private industry is low, especially for diseases primarily affecting low-income countries^84^. Most vaccine development to date, including COVID-19 vaccines, has been massively subsidized by government, academic, and philanthropic entities. Indeed, Phase I clinical trials of the first-in-human Ct vaccine, CTH522, were, in part, funded by the European Commission. The U.S. National Institutes of Health have also released numerous funding opportunities for research leading to a Ct vaccine. Yet, as identified earlier, lower patient and provider buy-in for vaccines against antibiotic-curable STIs remains a barrier to their production by high-income countries, whose concentration of manufacturers renders resource-poor countries lacking vaccine sovereignty^85^.

Advancing a Ct vaccine that can be effectively delivered to all people in need becomes a matter of social justice. Several models have shown that an efficacious Ct vaccine would be cost-effective^86,87^. Given the myriad disincentives for private development, this will likely require funding from government and/or non-profit entities. Emphasis must also be placed on development of Ct vaccines that require no special handling or storage conditions to ensure that they can be adequately stored, distributed, and administered where they are needed most.

## Patient-centered design early in preclinical development will improve vaccine acceptance

Traditionally, issues of vaccine acceptance, delivery, and cost are considered at late stages of vaccine development, often at the point of human clinical trials or after. However, as we have observed recently with the COVID-19 vaccines, these issues have an enormous impact on the successful roll out of vaccination campaigns. And, indeed, these issues are directly related to concerns of equity and justice. For instance, the fact that the COVID-19 vaccines utilized a new vaccine approach meant that vaccine hesitancy was heightened in the population. We propose that consideration of these issues at the earliest stages of pre-clinical vaccine development can help avoid waste of resources and ensure successful Ct vaccine rollout when the time comes.

It is becoming more commonplace to undertake a patient-centered approach to research and include patient knowledge into research design. After all, patients are experts in their own experiences and the needs of their communities. Indeed, there is a movement to include patients in clinical trials to ensure greater success^88^. Patients may help shape research questions and appropriate outcomes to be measured, vet questionnaires and interview questions for appropriateness, and provide input in research instruments to be used^88^. Without patient involvement, the research conducted may address less meaningful research questions, exhaust valuable resources, and bring forward solutions in which patients are not interested^88^.

A successful example of patient input guiding research is found in Paradise, et al., who describe how Cambridge Health Alliance was able to use patient input to design a disease management program for chronic obstructive pulmonary disease (COPD)^89^. By reviewing online patient communities, qualitative literature published on the lived experience of patients with COPD, and undertaking targeted patient interviews with clinician input, the researchers found that their disease management program should include five key elements: tobacco cessation, pharmacist referral for inhaler teaching and rescue pack prescriptions, pulmonologist referral for medication optimization and pulmonary rehabilitation, mental health screening and treatment, and attention to advanced care planning and palliative care services^89^. A similar approach could be taken to understand the patient priorities surrounding a Ct vaccine. This may include acceptable number of doses, route of administration, cost, and perceived appropriate age of vaccination.

The Food and Drug Administration has begun considering patient experiences as well through the use of patient-reported outcomes (PROs), with an argument that patients are uniquely qualified to report this context of vaccine development^90^. PROs often measure experiences of disease-related symptoms, disease impact, health-related quality of life related to the disease, and the impact of an intervention (like a vaccine) on a patient^90^. This may be through questionnaires, symptom diaries, and validated instruments to assess quality of life^90^. However, PROs are typically implemented during a clinical trial. We argue that patient input is valuable before a vaccine or intervention reaches this late stage.

We propose that Ct vaccine scientists should engage with community partners and researchers with expertise in community-engaged and participatory research to define the parameters needed for a successful Ct vaccine. Studies of key stakeholders (including young adults, parents and guardians of children in the proposed vaccination age, and medical practitioners) to address questions about vaccine route of administration, number of doses, vaccine side effects, marketing, and other concerns will help inform pre-clinical vaccine efforts. Indeed, several groups have already begun conducting such studies^91–93^, providing a foundation for future research that can help guide Ct vaccine design.

Finally, regarding marketing, there may need to be gender-specific interventions undertaken with consistent messaging focused on male vaccination. As demonstrated in a scoping review highlighting barriers to HPV vaccination in men, men commonly lack awareness of HPV itself and the vaccine, resulting in an underestimation of their own risk for infection^94^. Indeed, among male undergraduates aged 18–26, nearly one-fourth believed that men could not contract HPV and that HPV only affects women^95^. Yet, healthcare professionals continue to play an integral part of educating and promoting health maintenance among men^94^. Likewise, a systemic review and meta-analysis found that both healthcare professional recommendations for vaccination and perceived vaccine benefits were the most influential correlates of acceptability among men^96^. Newman et al. conclude that public health campaigns that promote positive HPV vaccine attitudes and awareness of HPV risk in men could help to support vaccine acceptability among this group^96^. There may also be community-based approaches to consider to ensure equity among racial groups. Indeed, nearly a decade after HPV licensure, data from the Health Information National Trends Survey revealed that Non-Hispanic Blacks were 33% and 44% less likely to have heard of HPV or the HPV vaccine, respectively, and Hispanics were 27% and 53% less likely to have heard of HPV or the HPV vaccine, respectively, as compared to non-Hispanic whites^97^. A considered and thoughtful approach to public health campaigns should be coupled with a Ct vaccine rollout that would effectively encourage those eligible, particularly men and minoritized groups, to complete vaccination using shared knowledge.

## Conclusion

After decades of research, we are inching closer to human efficacy studies of Ct vaccines. Indeed, the first-in-human safety trial for a Ct vaccine was recently conducted in a small group of women^23^, and more vaccines are in development. Here, we have examined some of the challenges of Ct vaccine development through a lens of equity, justice, and patient-centered values. We argue that protecting against multiple serotypes with Ct vaccines is an issue of equity; that designing a Ct vaccine for males and females is an issue of shared gendered responsibility for STI prevention; that studies of Ct infection at all susceptible anatomic sites will promote inclusion of all people in the benefit of vaccination; and that a patient-centered and feasibility approach to vaccine design early in pre-clinical development of Ct vaccines will lead to successful roll-out of vaccination campaigns later. How can this be done? First, the field needs more information now on the acceptability of a variety of vaccine approaches for the potential target populations. This should include studies of men and women, parents of children who are eligible for HPV vaccination, and adolescents. Questions regarding acceptability of vaccination routes, number of vaccinations, acceptable side-effects can be answered now with appropriately designed studies. This can provide essential information to guide prioritization of vaccine design that will be acceptable for target populations. Second, in anticipation of vaccinating men and women and protecting at various anatomic sites, the field needs appropriate animals models of anorectal, oropharyngeal, and male urogenital tract infection in order to test vaccine efficacy. Clinical endpoints for human trials also need to be considered, especially for non-female reproductive tract infection. More studies need to be conducted in order to inform future clinical studies and ensure equity and justice. Our proposal is not without drawbacks—indeed vaccine development is challenging, and it may appear impossible to add these additional constraints on vaccine design when finding an efficacious Ct vaccine is already taking decades of research. However, if the goal of a Ct vaccine is to prevent Ct infection and the associated morbidity, then we should consider everything that will impact that goal, while ensuring equity and justice.
